# Spectral imaging of normal, hydrated, and desiccated porcine skin using polarized light

**DOI:** 10.1117/1.JBO.27.10.105001

**Published:** 2022-10-12

**Authors:** Ben E. Urban, Steven L. Jacques, Hrebesh M. Subhash

**Affiliations:** a Colgate-Palmolive, Global Technology and Design Center, Clinical Methods Development Laboratory, Piscataway, New Jersey, United States; b University of Washington, Department of Bioengineering, Seattle, Washington, United States

**Keywords:** imaging, tissue, polarization

## Abstract

**Significance:**

Spectroscopic and structural imaging of tissue layers is important for investigating tissue health. However, investigating superficial tissue is difficult using optical imaging, due to the convolved absorption and backscatter of light from deeper layers.

**Aim:**

This report investigates the effects of hydration and desiccation of *ex vivo* porcine skin on the reflectance of polarized light at different wavelengths (light-emitting diodes).

**Approach:**

We developed a spectroscopic polarized imaging system to investigate submicron changes in tissue structures. By separating polarized from depolarized backscattered light, submicron structural changes in subsurface and deeper tissue layers can be separated and monitored.

**Results:**

The results demonstrate that (1) polarized light reflectance is about 2%, consistent with ∼6 scattering events, on average; (2) there was little wavelength dependence to the reflectance of polarized light; (3) increased hydration leads to a modest increase in total reflectance (from 0.8 to 0.9), whereas desiccation had little effect; however, hydration did not affect polarized reflectance, but desiccation slightly lowered polarized reflectance.

**Conclusions:**

Higher scattering from the reticular dermis was likely due to swelling of collagen fiber bundles in the dermal layers, which increased fibril spacing. The epidermal skin surface showed little change due to the stratum corneum resisting desiccation and maintaining hydration.

## 1 Introduction

Optical properties of tissue divulge information about tissue health and chromophore concentrations.^1^^–^^4^ For skin, the tissue is composed of multiple layers, with each layer having a specific function.^5^^–^^8^ The deeper reticular dermis layer of tissue is composed of mostly water and collagen and provides mechanical support for the skin. The superficial layers of the skin are the upper papillary dermis and the epidermis, where skin pathology most often arises. An outer layer, the stratum corneum, is the main protective barrier of the body for preventing dehydration and protects against bacterial infections and exogenous chemicals. The underlying reticular dermis provides mechanical support for the superficial layers.

Polarized spectral imaging is a technique capable of distinguishing deeply scattered photons and superficially scattered photons at multiple wavelengths.^9^^–^^14^ The population of photons scattered by only one or a few scattering events from superficial tissue layers, the subdiffuse scatter, partially retains the orientation of the incident polarized light. The population of multiply scattered photons backscattered from the deeper tissue is depolarized. Using horizontally and linearly polarized illumination (H), the copolarized (HH) and cross-polarized (HV) reflectance images are acquired, yielding a difference image, S=HH−HV, that is due to superficial subdiffuse scatter, and a total reflectance image, S+D=HH+HV, where D is the deep tissue scatter image:

$$\text{co-polarized}=\mathrm{HH}=\frac{1}{2}D+S\;\text{cross-polarized}=\mathrm{HV}=\frac{1}{2}D\;\mathrm{superficial}=\mathrm{HH}\text{-}\mathrm{HV}=S=Q\;\mathrm{total}=\mathrm{HH}+\mathrm{HV}=S+D=I$$

where I and Q are the first two elements in the $[IQUV{]}^{T}$ Stokes vector that describes the polarization state of reflectance.

This paper reports on the ability of hydration and desiccation to change the I and Q reflectance of the skin. Hydration is expected to swell collagen fibers and increase the bandgap between fibrils, which increases the water/protein interfaces within the fiber that scatter more isotropically, and hence reflectance increases.^15^^–^^22^ Desiccation is expected to condense the fibrils within collagen fibers, which reduces such isotropic scatter, and hence reflectance decreases. But how strong are these hydration/desiccation effects? A study on *ex vivo* porcine skin is presented.

## 2 Materials and Methods

### 2.1 Experimental Setup

**Fig. 1:**
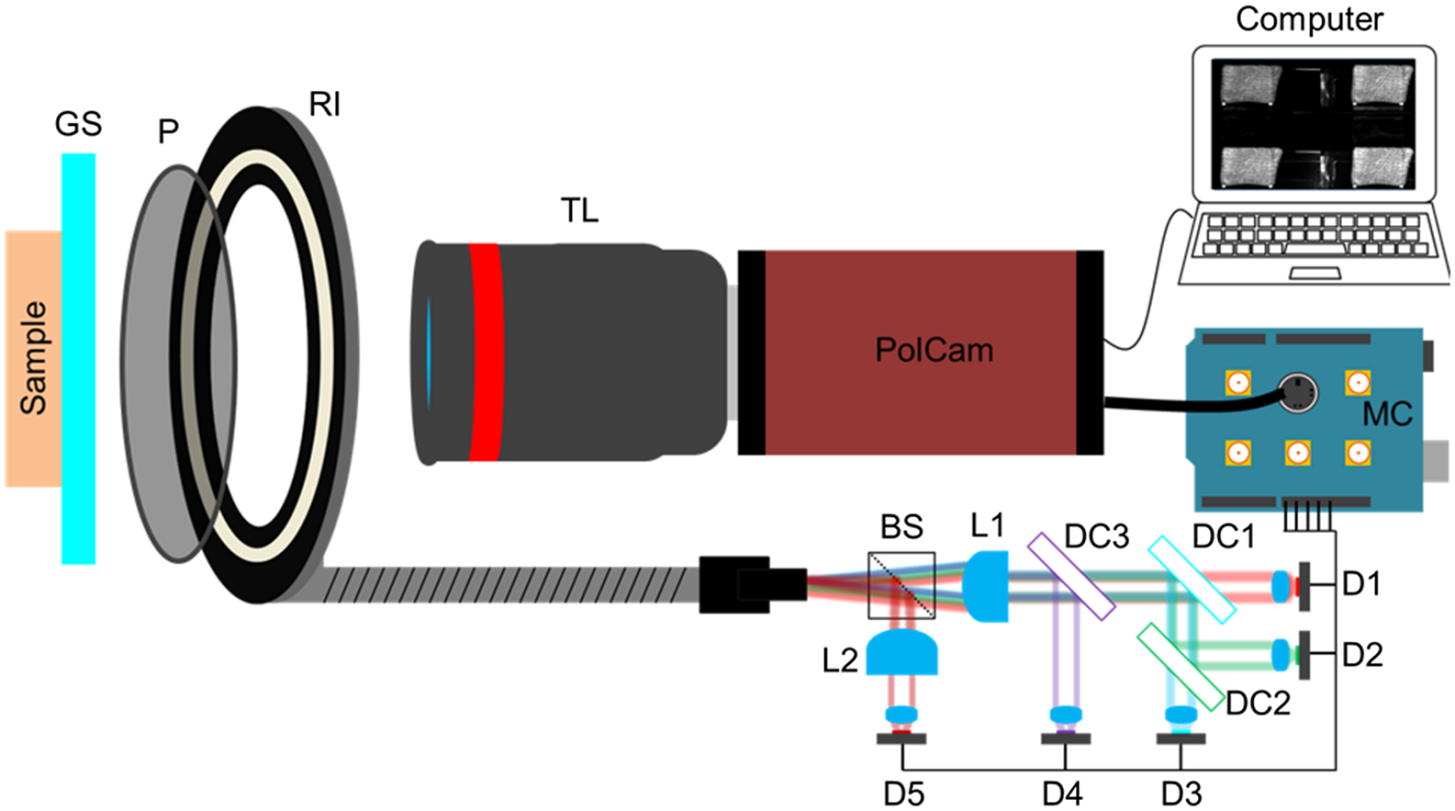
Simplified system schematic. Light from five LEDs (D1 to D5) was aligned into a single path using dichroic mirrors (DC1 to DC3) and a beam-splitter (BS). The light was focused using lenses (L1 and L2) into a light guide attached to a ring illuminator (RI) with a polarizer (P). A microcontroller (MC) was used to multiplex the LEDs and trigger the polarization camera (PolCam). Illumination light passed through a glass slide (GS) before being incident on the sample. Backscattered light was collected through a telescopic lens (TL) before being imaged by the camera.

Figure 1 shows the schematic of the experimental setup. Light from five spectrally unique LEDs (LED4D067, Thorlabs; M700L4, Thorlabs) (405 nm, FWHM 20 nm; 490 nm, FWHM 24 nm; 590nm, FWHM 14 nm; 660 nm, FWHM 17 nm; 700 nm, FWHM 20 nm) was combined into a single path using dichroic mirrors and a beam-splitter (BS019, Thorlabs). The light was then coupled into a fiber optic light guide with a ring-shaped illumination head (Schott, A08600). The coupled light passed through the ring illumination head with a diameter of 115 mm before transmitting through a linear polarizer (Schott, A08615). The polarized light was incident on a glass slide at ∼45  deg off the normal to the surface, which directed the specular reflection away from the imaging camera. The distance from the ring illuminator to the glass slide was adjusted to be ∼60  mm. The illumination profile was carefully adjusted to give near-uniform illumination over the imaging field of view, which was perpendicular to the camera-sample axis. The field of view was 67.5  mm×56.5  mm (horizon × vertical). Polarized light that passed through the glass side was delivered to a tissue sample that was coupled to the glass using silicone oil. Backscattered light from the tissue was collected using a telescopic lens (NMV-25M1, Navitar) and focused onto a polarization camera (CS505MUP, Thorlabs) with four polarization orientations (0 deg, 45 deg, −45  deg, and 90 deg), where 0 deg (HH) and 90 deg (HV) camera channels had copolarized and cross-polarized orientations with respect to the incident light, respectively. Image acquisition with the five illumination wavelengths was synchronized using a custom program written in Arduino IDE. Image integration time was set to 100 ms for each wavelength. The LEDs were turned on 7 ms before the camera acquisition to stabilize illumination. The total image acquisition time was 530 ms (∼2  Hz).

### 2.2 System Calibration

**Fig. 2:**
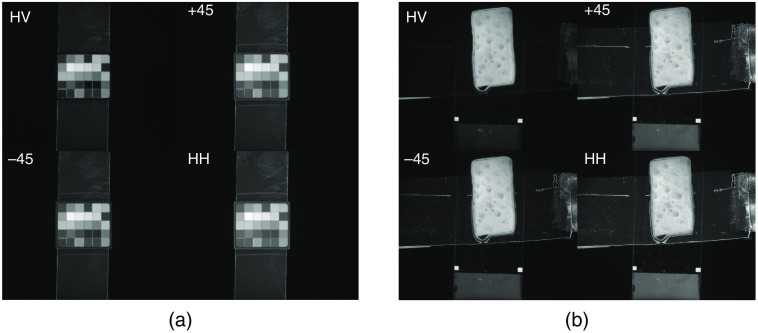
Raw image of the (a) Macbeth color chart and (b) porcine tissue corrected by 1/γ.

The system calibration was done using custom routines written in MATLAB (2019b, MathWorks). Figure 2 show the raw image of a Macbeth color chart and an example porcine sample. First, a γ correction was applied to all raw images to undo the automatic decompression imposed by MATLAB (figures had been saved as tiff files with no γ correction, but MATLAB assumed that there was γ correction and automatically decompressed). The corrected image is summarized as corrected image A(λ):

$$A(\lambda )=\mathrm{image}(\lambda {)}^{1/\gamma },$$ (1)

where γ=2.2, 1/γ=0.4545. The correction was applied to the raw HH and HV images at each wavelength (λ), such that

$$A(\lambda {)}_{\mathrm{HH}}=\mathrm{HH}(\lambda {)}^{1/\gamma },$$ (2)

$$A(\lambda {)}_{\mathrm{HV}}=\mathrm{HV}(\lambda {)}^{1/\gamma }.$$ (3)

Then the intensity ($A_{I}$) and difference image ($A_{Q}$) were calculated as

$$A(\lambda {)}_{I}=A(\lambda {)}_{\mathrm{HH}}+A(\lambda {)}_{\mathrm{HV}},$$ (4)

$$A(\lambda {)}_{Q}=A(\lambda {)}_{\mathrm{HH}}-A(\lambda {)}_{\mathrm{HV}}.$$ (5)

**Fig. 3:**
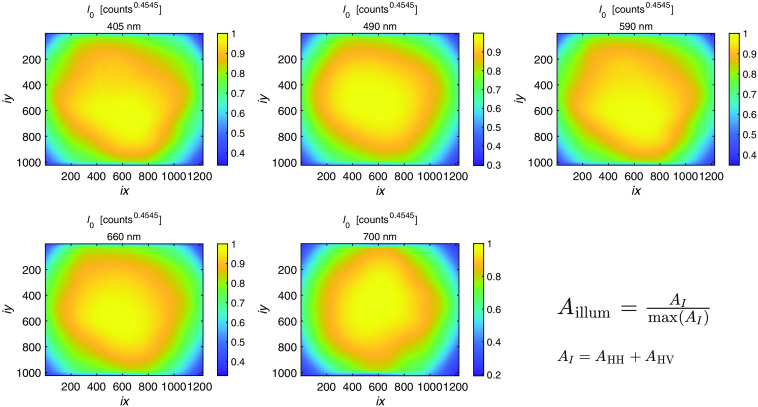
Relative nonuniformity of illumination for the five LEDs on a neutral reflection standard. The captured images were normalized by the maximum pixel value, defining the correction factor.

After applying the γ correction, the nonuniform illumination of the system was corrected. The illumination profile of each LED was captured by imaging a neutral reflectance standard, as shown in Fig. 3. The images were normalized by the maximum pixel value to create a uniformity-of-illumination image, $A_{\mathrm{illum}}$, for all five LEDs:

$$A(\lambda {)}_{\mathrm{illum}}=A(\lambda {)}_{I}/\max(A(\lambda {)}_{I}).$$ (6)

The normalization corrects the nonuniform illumination and sets the pixel values between 0 and 1, yielding an illumination corrected intensity image ($M_{I}(\lambda )$) and a difference image ($M_{Q}(\lambda )$):

$$M(\lambda {)}_{I}=\frac{A(\lambda {)}_{I\;\mathrm{sample}}}{A(\lambda {)}_{\mathrm{illum}}},$$ (7)

$$M(\lambda {)}_{Q}=\frac{A(\lambda {)}_{Q\;\mathrm{sample}}}{A(\lambda {)}_{\mathrm{illum}}},$$ (8)

where $A(\lambda {)}_{I\;\mathrm{sample}}$ is the corrected sample intensity image at wavelength λ and $A(\lambda {)}_{Q\;\mathrm{sample}}$ is the corrected difference image at wavelength λ.

**Fig. 4:**
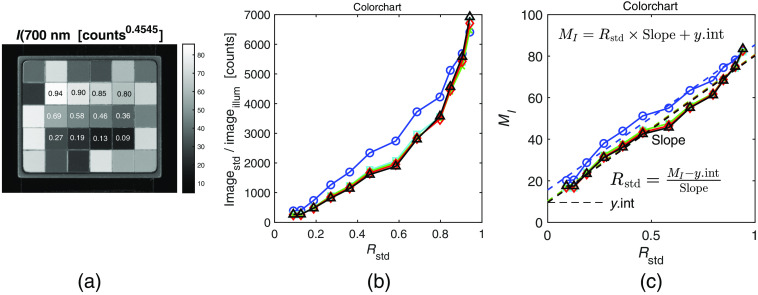
Calibration of the camera response to the five LEDs. (a) The MacBeth color chart used for correcting the polarization camera at each wavelength. (b) The uncorrected response of the polarization camera to each of the five LEDs. (c) The corrected response to the LEDs using the 12 neutral tiles (black to white) of the color chart is shown in (a).

The 12 neutral colors (black–gray–white) of a Macbeth color chart (ColorGauge Micro Target, Image Science Associates, $R_{\text{std}}=0.0902$, 0.1268, 0.1882, 0.2706, 0.3621, 0.4588, 0.5843, 0.6863, 0.7974, 0.8471, 0.9046, and 0.9412) were used to calibrate $M_{I}$ and $M_{Q}$ into reflectance values, as shown in Fig. 4. The mean values of the neutral tiles were acquired by averaging a 20×20-pixel region of each of the tiles. The uncorrected ($\mathrm{image}/{\mathrm{image}}_{\mathrm{illum}}$) and corrected ($M_{I}$) values were plotted versus $R_{\text{std}}$ for each LED wavelength. The corrected $M_{\text{std}}$ versus $R_{\text{std}}$ showed a linear relationship having its own slope and y-intercept ($y_{\text{int}}$). Subsequently, the slope and y-intercept were used to calibrate the reflectance scale of the camera image as

$$R=\frac{M-y_{\mathrm{int}}}{\mathrm{slope}},$$ (9)

where M is the intensity corrected image and R is the calibrated reflectance image.

### 2.3 Sample Preparation and Imaging Acquisition

**Fig. 5:**
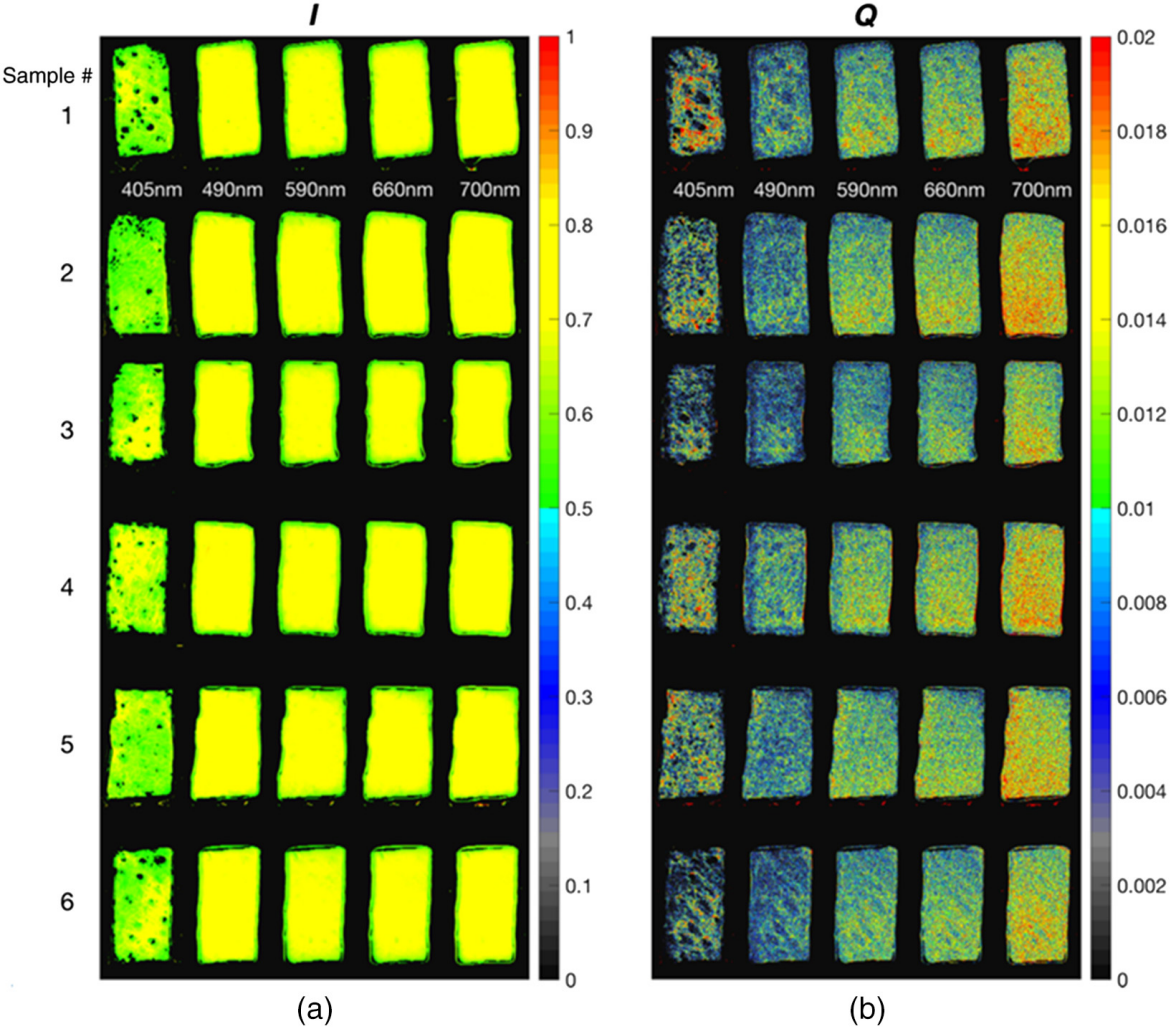
(a) I dermal tissue images acquired at the five different wavelengths for the control condition. (b) Same as (a) but for Q images. The I image gives information about the bulk tissue, whereas the Q image contains only superficial tissue information. Inhomogeneity can be seen across samples, especially for the Q images.

Porcine tissue slices, purchased from an abattoir supplier (Animal Technologies, Inc.; Tyler, Texas), were used for hydration and desiccation experiments. The porcine tissue was freshly acquired from an albino pig. Hair and fat tissue was removed from the tissue slices. The tissue was then cut into $25\times 12\;{\mathrm{mm}}^{2}$ with an approximate thickness of 4 mm before immediately being shipped overnight on ice for imaging. To determine the effect of hydration and desiccation on tissue scattering, tissue samples were placed in three different environments and labeled as (1) control, (2) hydrated, or (3) dry. The control tissue samples were created by placing porcine tissue slices in a container for 1 h. The container had a chamber that contained small amounts of water to mimic environmental humidity. The hydrated tissue samples were created by immersing separate tissue slices in distilled water for 1 h. The dehydrated tissue samples were created by sandwiching porcine tissue slices between two blotting pads for 1 h to remove water. After 1 h, the samples were submerged in silicone oil to prevent changes in water content. For imaging, samples were coupled to a glass slide using the silicone oil as an interface. The front and back of the samples were imaged using the polarized spectral imaging system. Images acquired from tissue were processed to separate superficial layer scattering from deep-tissue scattering using routines written in MATLAB. Figure 5 shows calibrated reflectance images of the six reticular dermis (rear surface of *ex vivo* sample) control samples. The intensity I and reflectance Q are ∼60% to 80% (STD 7.8) and 0.6% to 2% (STD 0.3) of the incident light, respectively. Sample-to-sample deviation in I and Q is expected due to the inhomogeneity of tissue. Notably, reflectance Q values for the tissue sample are within the range of the values calculated for the neutral tiles of the color chart; however, the 405 nm tissue Q values were only slightly above 0.6%.

## 3 Results

**Fig. 6:**
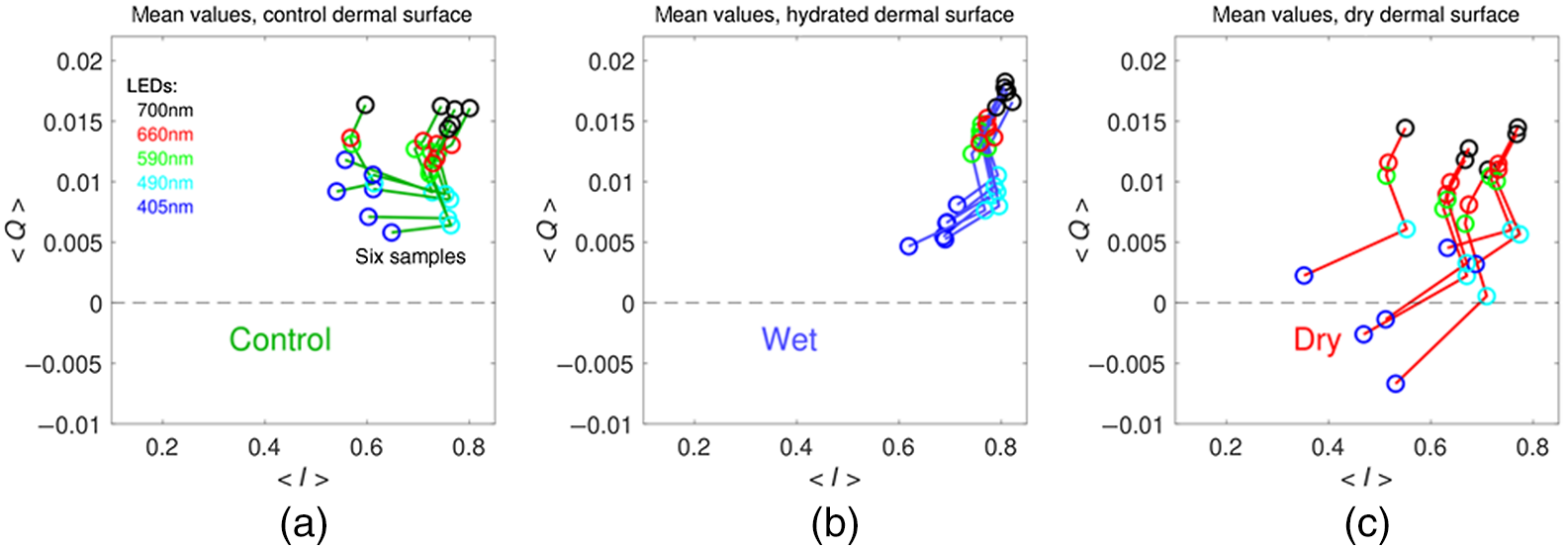
Porcine dermal tissue Q versus I plots at the different wavelengths for the (a) control, (b) hydrated, and (c) dry sample environments. The wavelength of illumination is indicated by the color of the open circles: blue −405  nm, cyan −490  nm, green −590  nm, red −660  nm, and black −700  nm.

The mean dermal I and Q measurements for the different tissue environments and wavelengths are plotted in Fig. 6. Mean values were acquired from the pixel values contained in a bounding box placed around the tissue in MATLAB. Differences in tissues in the same environmental conditions were present, but a distinction between I and Q for different environmental conditions was still observable. In Fig. 6(c) (dry), a few of the Q values are negative, which is likely due to a slight mismatch in the HH and HV measurements. Rather than attempting to correct this error, the data are presented as recorded to avoid any extra data manipulations.

**Fig. 7:**
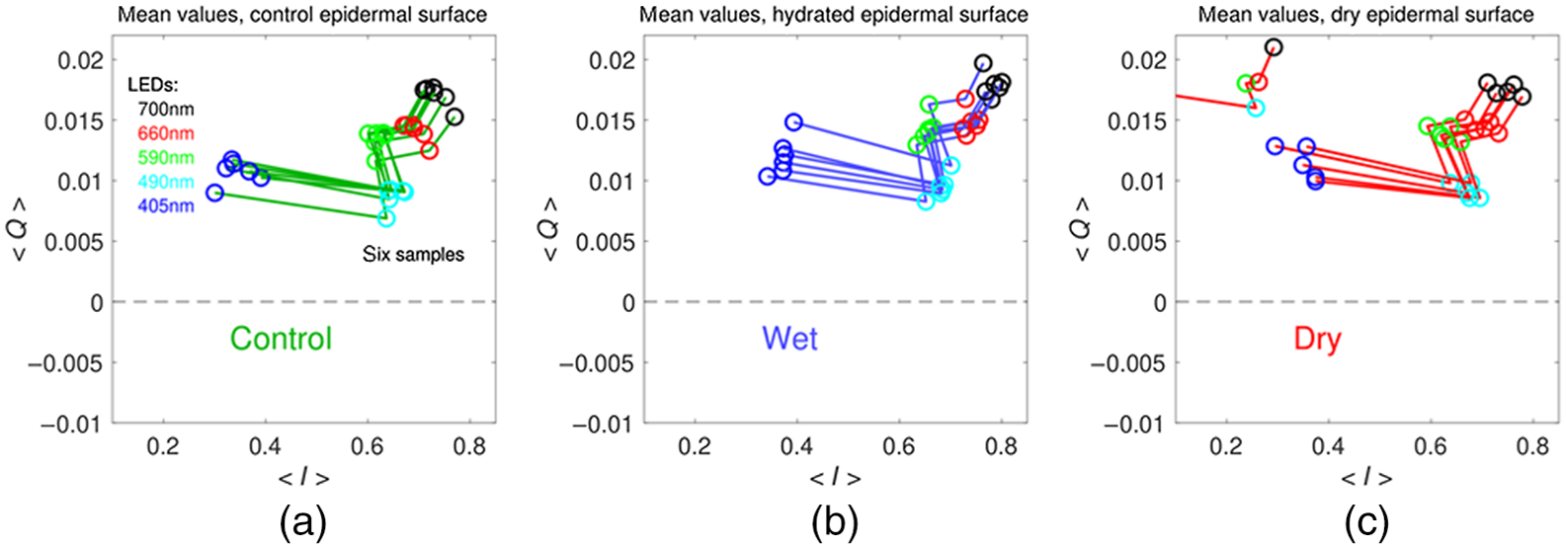
Porcine epidermal tissue Q versus I plots at the different wavelengths for the (a) control, (b) hydrated, and (c) dry sample environments. The wavelength of illumination is indicated by the color of the open circles: blue −405  nm, cyan −490  nm, green −590  nm, red −660  nm, and black −700  nm. One dry epidermal sample was deviant, and one datum extended off the graph to Q=0.0154 and I=0.0173.

Figure 7 shows I and Q measurements of the epidermal side of the tissues at different wavelengths. Sample-to-sample variation is observed, but a distinction between I and Q for the different environments is not clear.

## 4 Discussion

**Fig. 8:**
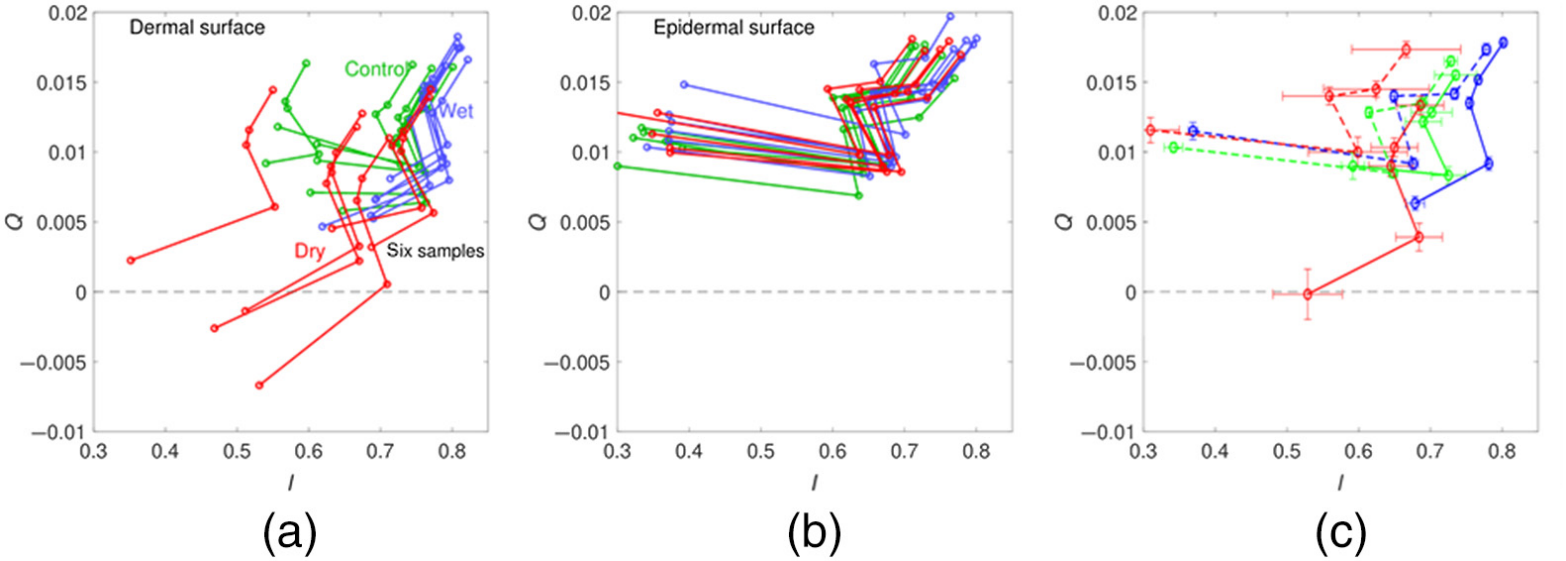
(a) Dermal surface Q versus I plot of all tissues in the different environments (green – control; blue – hydrated; red – dry). (b) same as (a) but for the epidermal surface. One dry epidermal sample was deviant, and one datum extended off the graph to Q=0.0154, I=0.0173. (c) Average of the dermal samples (solid lines) and epidermal samples (dashed lines). Error bars are standard error of the mean.

Figure 8 shows the backscattering trend for all environments and both tissue surfaces. For dermal tissue, the I signal increased with the increased hydration of the tissue by ∼10% (from 0.8 to 0.9). This is likely due to the separation of fibrils within collagen fibers, which yields more isotropic scatter from the very small fibers and hence more reflectance. The shorter wavelengths had a lower I than the longer wavelengths, presumably due to optical absorption by the residual blood, regardless of the environmental conditions.^23^ The mean dermal Q signal, which divulges subsurface scattering information, decreased with desiccation. The difference in the Q signal was more pronounced at the 405 nm wavelength for all three environmental conditions. The shorter wavelength is more sensitive to changes in the fibril packing within collagen fiber bundles.^24^^–^^26^ Therefore, the 405 nm Q images are more sensitive to changes in tissue hydration than images using longer wavelengths. In addition, depending on the penetration depth of water into the tissue, higher energy wavelengths may be more sensitive to scattering due to sampling shallower regions of the tissue. Comparing the dermal tissue results from the three environmental conditions shows that hydration increases the total reflectance (I≈0.80), whereas desiccation decreases both I and Q.

For epidermal tissue, both I and Q signals had little dependence on the environmental condition. The insensitivity of I and Q signals, despite the drastically different environmental conditions, is likely due to the stratum corneum resisting desiccation of the epidermal skin tissue surface and limiting water penetration.^5^^–^^7^^,^^27^ Therefore, changes in collagen fibril packing would not be as significant. The hour-long hydration of the skin samples apparently did not diffuse sufficiently to affect the upper skin layers that yield topical reflectance. Additionally, water diffusion from the dermal side did not seemingly penetrate deep enough into the tissue to significantly change I in measurements from the epidermal side. Another possibility is that collagen fibers near the stratum corneum were already near their water peak size due to the stratum corneum maintaining hydration. If the fibers in the layer were already near peak size in the control sample and the stratum corneum protected against desiccation, there may have been an insignificant change in the size of bulk fiber spacing for the subsurface epidermis layer. Additionally, though the stratum corneum can incorporate water, the layer contains very little collagen and is very thin.^6^^,^^27^^–^^30^ Therefore, water content of the stratum corneum is not expected to significantly contribute to depolarization of incident light.

## 5 Summary

HH and HV images were used to separate the superficial and deeper tissue layers. Tissues were illuminated at different wavelengths, and the I and Q images were used to investigate how hydration and desiccation of skin affect polarized light. We found that increased hydration leads to increases in total reflectance, whereas desiccation had little effect. Furthermore, polarized reflectance did not change during hydration, but desiccation slightly lowered polarized reflectance. This investigation is potentially useful for mapping the hydration of bulk and subsurface tissue over large areas.

## Code, Data, and Materials Availability

The code and data can be obtained by contacting the authors.
